# Soil Microbial Communities Associated with Three Arctic Plants in Different Local Environments in Ny–Ålesund, Svalbard

**DOI:** 10.4014/jmb.2208.08009

**Published:** 2022-09-20

**Authors:** Deokjoo Son, Eun Ju Lee

**Affiliations:** 1 College of Education Department of Science Education, Dankook University, Gyeonggi-do 16890, Republic of Korea; 2 Biological Sciences, Seoul National University, Seoul 08826, Republic of Korea

**Keywords:** Soil pH, Proteobacteria, Acidobacteria, spatial heterogeneity, high Arctic

## Abstract

Understanding soil microbial community structure in the Arctic is essential for predicting the impact of climate change on interactions between organisms living in polar environments. The hypothesis of the present study was that soil microbial communities and soil chemical characteristics would vary depending on their associated plant species and local environments in Arctic mature soils. We analyzed soil bacterial communities and soil chemical characteristics from soil without vegetation (bare soil) and rhizosphere soil of three Arctic plants (*Cassiope tetragona* [L.] D. Don, *Dryas octopetala* L. and *Silene acaulis* [L.] Jacq.) in different local environments (coal-mined site and seashore-adjacent site). We did not observe any clear differences in microbial community structure in samples belonging to different plant rhizospheres; however, samples from different environmental sites had distinct microbial community structure. The samples from coal-mined site had a relatively higher abundance of Bacteroidetes and Firmicutes. On the other hand, Acidobacteria was more prevalent in seashore-adjacent samples. The relative abundance of Proteobacteria and Acidobacteria decreased toward higher soil pH, whereas that of Bacteroidetes and Firmicutes was positively correlated with soil pH. Our results suggest that soil bacterial community dissimilarity can be driven by spatial heterogeneity in deglaciated mature soil. Furthermore, these results indicate that soil microbial composition and relative abundance are more affected by soil pH, an abiotic factor, than plant species, a biotic factor.

## Introduction

Microbial community structure and activity in glacier forelands are strongly affected by the interactions between physical and chemical variables [1] such as soil type and nutrition [2-4], as well as plant species, which have unique root exudation patterns [5, 6]. Furthermore, land-use types can shape soil microbial communities by modulating community structure [7]. These soil microbial communities modulate vegetation structure during colonization [8], and plants interact with the microbial community through root exudates and litter inputs, which are particularly important in nutrient-poor soil [9]. Rhizodeposition, microbial activity, redox reactions, root nutrient uptake, and CO_2_ production all distinguish pioneer plant rhizosphere soil from bulk soil [10-12]. Understanding the spatial distribution of soil microbial communities is essential for determining how these communities contribute to biogeochemical cycling, plant colonization, and soil development in the Arctic region [8, 13, 14].

Soil microbial community diversity in the Arctic has been investigated for several decades, with recent research on microbial community structure and function enabled by advancements in genomics [8]. In particular, next-generation sequencing is an important technique in ecology to sequence multiple samples and explore bacterial species diversity, either by targeting the 16S rRNA gene or by directly sequencing genomic DNA or RNA [15, 16]. Several studies mainly use chronosequence methods that examine soil structure and development at a linear distance from glacier edges [8, 17, 18]. In addition, most examinations of plant succession in glacier forelands do not consider plant–associated bacterial communities [18, 19]. Little attention has been paid to pioneer plant–associated bacterial communities from nutrient-poor soils in the high Arctic [11, 20-22]. Therefore, the dominant vascular plant–associated bacterial communities and diversity are in need of further study throughout the area, from deglaciated spaces to the shoreline, where vegetation has developed.

Due to the effects of increasing temperature and precipitation, Arctic terrestrial ecosystems are facing rapid changes caused by sea ice declines, permafrost thawing, and glacial retreat [8, 23]. Deglaciation has created lakes, moraines and valleys [18] where microbes contribute soil development, vegetation structure and primary succession [24]. These landscape changes are evident, for one, in the Svalbard, Norwegian high Arctic. Historically, commercial coal mining began in Ny–Ålesund of the Svalbard archipelago in 1916 and continued until a large accident in 1962 halted operations in 1963 [25]. Heavy metals and other soil elements may still linger from coal waste materials and other remnants of mining operations, such as machinery and equipment [25, 26]. Therefore, the soil of Ny–Ålesund is influenced by various environmental factors such as glaciers, glacier water, seawater, plant establishment and coal-mined sites.

In this study, we focused on characterizing the soil bacterial communities associated with plant species and local environments in a mature deglaciation stage [8, 27] in Ny–Ålesund, Svalbard. We hypothesized that bacterial community structure and diversity as determined by 16S rRNA gene pyrosequencing would differ (1) between the rhizospheres of three Arctic plants, or (2) in different local environments (coal-mined and seashore-adjacent sites). Samples’ soil chemical characteristics were also compared to account for differences in bacterial community diversity and structure.

## Methods

### Site Description

The study site is located in Ny–Ålesund (78°55′ N, 11°55′ E) in the largest island of Norway’s Svalbard archipelago: Spitsbergen. Ny–Ålesund is part of the high Arctic region, where temperature and annual precipitation have been on the rise. From 1995 to 1998, the annual mean temperature was -5.5°C and the total annual precipitation (snow or rain) was 362 mm in this area [28]. However, from 2011 to 2015, the annual mean temperature rose to ­3.2°C and total annual precipitation rose to 490.5 mm (http://en.tutiempo.net/climate/ws-10070.html). Unusually, the total annual precipitation in 2016 was 1,003 mm (http://en.tutiempo.net/climate/ws-10070.html). While the mean temperature in July 2015, when this study was conducted, was higher than average July temperatures (5.8°C) at 6.5°C, precipitation was lower than usual (32.0 mm) at 13.2 mm (https://www.yr.no/). The region’s snow-free duration lasts from June to August [29].

**Fig. 1:**
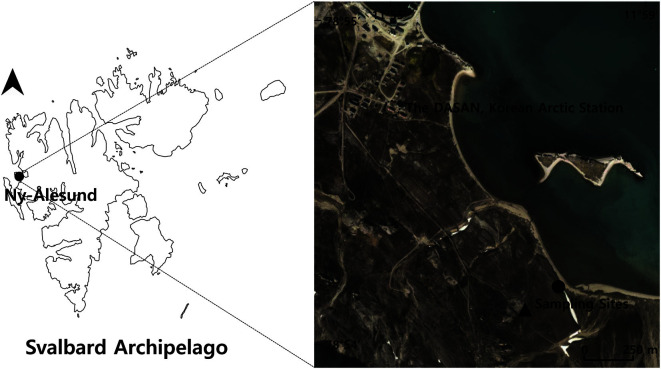
Study area and sampling sites. The circle (●) represents the seashore-adjacent site and the triangle (▲) represents the coal-mined site.

The sampling sites were 1.3 km south and east of the Korean Dasan Station (Fig. 1). One site was located 30 m from the seashore (78°54′59′′ N, 11°58′21′′ E), while the other site was near coal-mined land (78°54′53′′ N, 11°57′58′′ E). The two sites were located 200 m apart and the difference in elevation between the sites was about 12 m. These sites are older, deglaciated sites outside of the glacier moraine, where the soil is well developed to support vegetation [8]; however, these sites have heterogeneous environments.

### Sample Collection and Soil Chemical Analysis

**Fig. 2:**
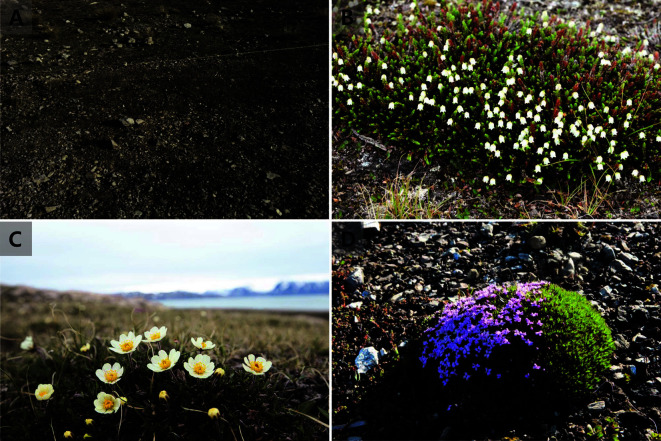
Pictures of bare soil (**A**), *C. tetragona* (L.) D. Don (**B**), *D. octopetala* L. (**C**) and *S. acaulis* (L.) Jacq (**D**).

Three vascular plants were especially abundant at sampling sites and broadly distributed in the arctic and alpine tundra: *Cassiope tetragona* (L.) D. Don, *Dryas octopetala* L. and *Silene acaulis* (L.) Jacq. (Fig. 2). *C. tetragona* and *D. octopetala* are circumpolar evergreen dwarf shrubs that form dense compressed mats [30, 31] and stand up to 20 cm in height [32]. Increases in the growth and abundance of shrubs in many tundra ecosystems, likely due to climate warming, are among the most prominent recent ecological changes to this environment [33]. Consequently, there is a growing need to understand these two shrub species and their associated soil microbes. *S. acaulis* is an evergreen perennial cushion plant. Individual cushions have a single taproot and grow by adding branch tips [34, 35]. The average area of a mat or cushion is 259 cm^2^ (standard error [SE] = 23, *n* = 116) for *C. tetragona*, 313 cm^2^ (SE = 29, *n* = 94) for *D. octopetala*, and 118 cm^2^ (SE = 10, *n* = 206) for *S. acaulis* (unpublished data). These three flowering plants are considered important because they can respond uniquely to climate, such as winter snowfall, the timing of summer snowmelt, and air temperature in the Arctic [36].

To collect soil for analysis of chemical characteristics and rhizospheric microbial communities, soil samples were taken from beneath each of the three previously mentioned vascular plants as well as from soil with no vegetation (bare soil) (0–5 cm depth) in two different environments (coal-mined site, seashore-adjacent site) in July 2015. The vascular plants plots were 2–3 m apart. Soil samples were stored in situ using Exgene Soil DNA mini kits (GeneAll Biotechnology, Korea). Bare soil was taken from three locations (upper left, middle, lower right) in a 1 × 1 m^2^ quadrat and then mixed thoroughly to produce a composite sample.

Soil electrical conductivity (EC) and pH (soil:distilled water, 1:5) were measured using a Eutech PC 2700 (EUTECH Instrument, Singapore). Organic matter content (OM) was analyzed by loss on ignition (combustion at 550°C for 4 h) [37]. Total nitrogen and total carbon were determined using an element analyzer (EA1110, CE Instruments, UK) at the National Instrumentation Center for Environmental Management of Seoul National University and total C and N were then used to calculate C:N ratios. Exchangeable cations Na, K, Mg, and Ca were extracted with 1 N ammonium acetate and measured using inductively coupled plasma–mass spectrometry (ICP-730ES, Australia). The dried and milled soil samples were heated with HNO3 and HCl (1:3) to extract heavy metals, which were analyzed using ICP-730ES based on the EPA 3052 method [38].

### DNA Extraction and Sequencing

Genomic DNA was extracted from 0.5 g of soil sampled from eight sites using Exgene Soil DNA mini kits (GeneAll Biotechnology) and following the manufacturer’s instructions. Extracted DNA was purified using Expin kits (GeneAll Biotechnology). For microbial community analysis, all samples were identified by amplification and sequencing of the hypervariable V3-V4 region of the 16S rRNA gene of bacteria [39]. Amplification was performed using an initial denaturation step at 95°C for 7 min, followed by 30 cycles at 95°C for 30 sec, 55°C for 30 sec and 72°C for 30 sec, and a final extension step at 72°C for 10 min [40]. 454 pyrosequencing was conducted using a Roche GS FLX pyrosequencer (Macrogen, Korea), following the manufacturer’s instructions.

### Sequence Processing and Analysis

To increase sequencing data quality, raw reads were processed to remove short reads (< 300 bp) and reads longer than the expected size of PCR products [41], using the mothur pipeline (http://www.mothur.org/) [42]. PCR chimeras that were unassigned and/or related to non-bacterial sequences were removed [43]. All sequences were identified using the Ribosomal Database Project (RDP) and taxonomically assigned based on RDP classifiers [41, 44]. Diversity indices, such as Shannon diversity, Simpson diversity, and the Chao1 estimator, were calculated using the mothur package [45] at a threshold of 97% sequence similarity [46]. A maximum likelihood phylogenetic tree was developed using MEGA6 [47] with 1,000 bootstrap replicates [41]. We used principal coordinates analysis to analyze the abundance of bacterial classes and to visualize differences in soil bacterial communities [48], using the “vegan” package [49] in R (https://cran.r-project.org/bin/windows/base/) [50]. A linear regression was conducted in R to determine the relationships between the relative abundance of four major prevalent phyla and soil pH. Soil chemical characteristics between coal-mined land and the sites close to the seashore were compared using a nonparametric Mann–Whitney U test [51].

## Results and Discussion

### Soil Chemical Characteristics

**Fig. 3:**
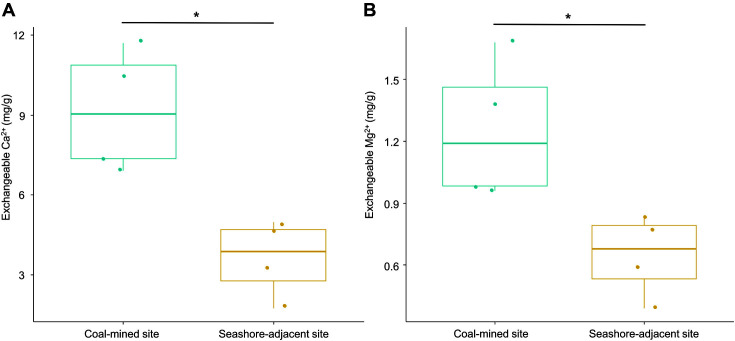
Boxplots depicting exchangeable Ca^2+^ (**A**) and Mg^2+^ (**B**). Points represent each sample. Asterisks indicate *p* < 0.05 in comparisons between coal-mined and seashore-adjacent sites, as determined by a Mann–Whitney U test.

Table 1 compares soil characteristics between sites (coal-mined and seashore-adjacent, *n* = 4), using a Mann–Whitney U test. The concentration of exchangeable Ca^2+^ and Mg^2+^ was higher in the coal-mined site (Ca^2+^ 9.18 mg/g; Mg^2+^ 1.25 mg/g) than in the seashore-adjacent site (Ca^2+^ 3.62 mg/g; Mg^2+^ 0.65 mg/g) (Fig. 3). Across samples, soil pH was weakly acidic (5.48–6.44); however, mean pH from the seashore-adjacent site (5.55 ± 0.07, mean ± SD) was lower than that of the coal-mined site (5.92 ± 0.36). Similar soil pH (5.0–6.0) in Svalbard has been reported by Lee *et al*. [52], who also proposed that soil pH is one of the strongest factors determining bacterial composition in the Arctic.

The average OM concentration was 8.23% in the coal-mined site and 7.55% in the seashore-adjacent site. These results concur with previous work that found an average 7.77% OM in the organic layer of Arctic tundra soils [52]. Across all samples except the bare soil sample from the coal-mined site, the C:N ratio ranged from 12.2 to 19.1. Kim *et al*. [18] also reported C:N ratios ranging from 13.2 to 21.3 in mature soil outside the glacier moraine in Ny–Ålesund. In addition, iron (Fe^2+^) ranged from 7.54 to 17.65 mg/g, which was lower than that reported by previous research (30.2 to 39.3 mg/g) on mature soils in Ny–Ålesund [8].

Though the small number of samples limits the ability to draw a direct conclusion, the sample of bare soil from the coal-mined site seems to be an outlier; among the samples in this study, this bare soil sample possessed the maximum concentrations for EC, OM, C:N ratio and Fe^2+^. The seashore was slightly lower in altitude (about 12 m) than the coal-mined site, where the presence of wild animals (*e.g.*, reindeer, Arctic fox) and vegetation cover was high. Reindeer are known to eat a wide breadth of vegetation, with their selection driven more by plant quantity than quality [53]. Therefore, the bare soil from coal-mined site might have been fertilized by plant litter or animal feces. Animal feces can be used as an important vector for the transfer of environmental elements because they contain high concentrations of metals and organic matter [54, 55]. In conclusion, despite limited sample sizes, we found small differences in soil characteristics between two local environments.

### Bacterial Community Structure

**Fig. 4:**
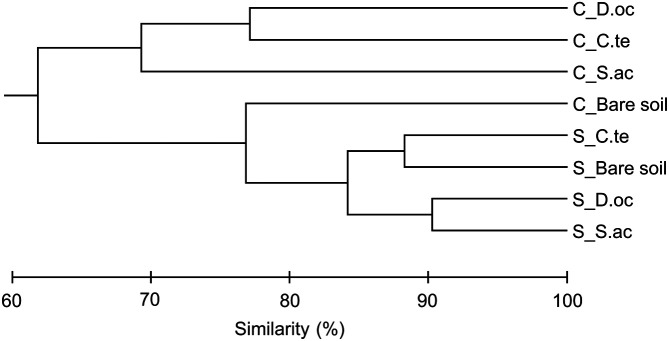
Cluster analysis of bacterial community structure using OTU abundance–based Bray–Curtis similarity coefficients. C, coal-mined site; S, seashore-adjacent site. C.te, *C. tetragona*; D.oc, *D. octopetala*; S.ac, *S. acaulis*.

**Fig. 5:**
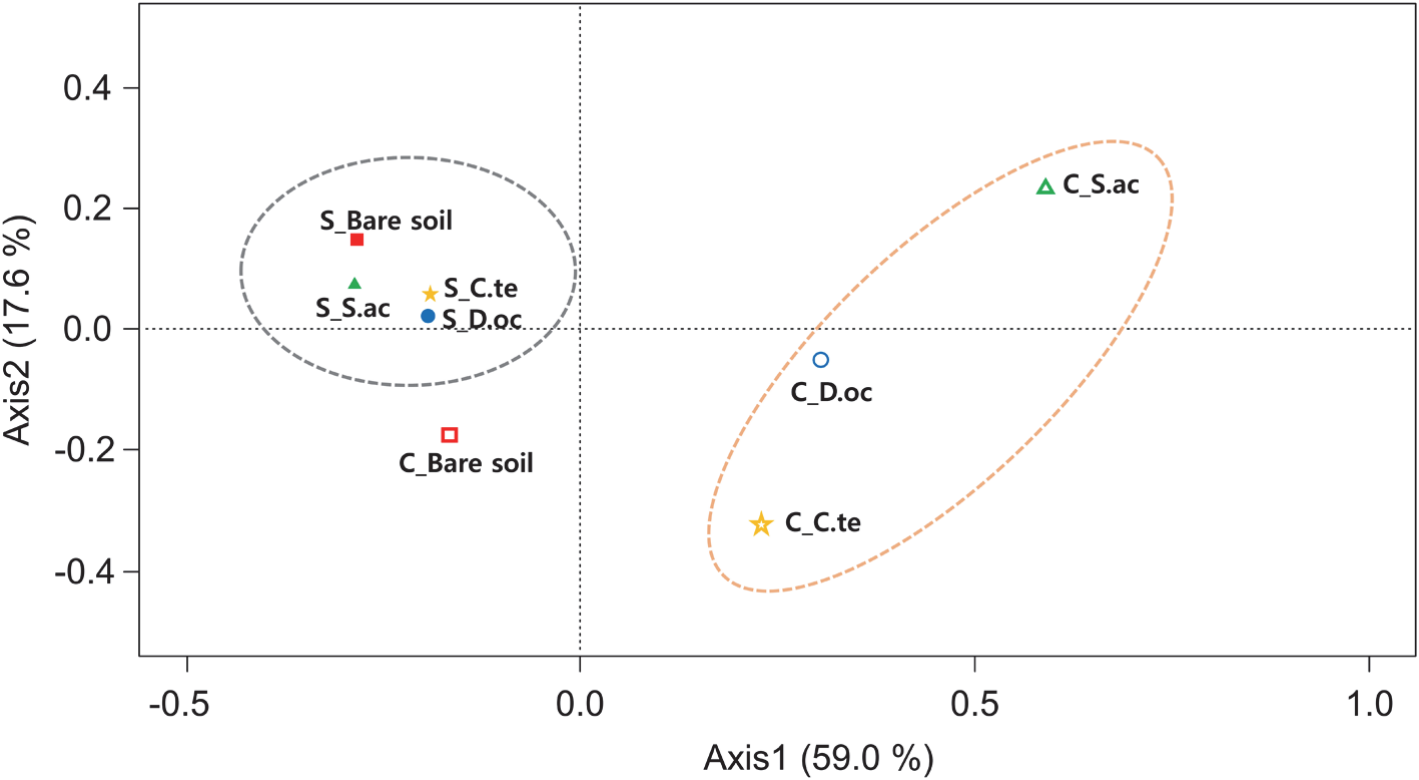
Principal coordinates analysis of soil bacterial communities at the class level from bare soil and three Arctic plant rhizospheres. C, coal-mined site; S, seashore-adjacent site. C.te, *C. tetragona*; D.oc, *D. octopetala*; S.ac, *S. acaulis*.

In total, 26,385 OTUs occurred with a frequency of at least 8 reads at the phylum level in the dataset. Bacterial community samples clustered into roughly two groups that corresponded well to the local environment from which they were sampled (Fig. 4). Similarly, bacterial samples clustered according to their local environment more so than by their associated plant species, except for bare soil from coal-mined site (C_Bare soil) (Fig. 5). Furthermore, the bacterial community of the seashore-adjacent samples clustered together more tightly and exhibited smaller variance than those from the coal-mined site. Surprisingly, the bare soil sample from coal-mined site clustered more closely with the seashore-adjacent samples. This is consistent with this sample’s distinct soil characteristics. Chu *et al*. [56] proposed that soil bacterial community structure is strongly influenced by vegetation type in the low Arctic tundra. Additionally, bacterial communities at the phylum level exhibited large differences in composition between shrub- and tussock-associated soils in Alaska [57]. Specific ecological niches in soil can be created by plants, which may select a particular combination of bacterial species and functional groups [20, 58]. Vegetation type can affect soil biotic and abiotic factors, which, in turn, influence the local soil microbial community [59].

However, similar to our results, bacterial communities in soils dominated by shrubs and grasses in a Finnish Arctic tundra did not differ at the phylum level and instead, soil pH had a major influence on microbial community composition [60]. According to Viitamäki *et al*. [59], soil microbial community composition and function in the sub-Arctic tundra can vary across soil properties and vegetation types. Therefore, although several studies have demonstrated the impact of vegetation on microbial communities in the Arctic, the magnitude of this influence varies by location [61].

**Fig. 6:**
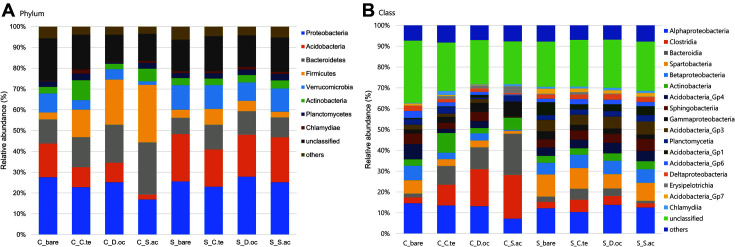
Relative abundance of sampled bacterial communities at the (**A**) phylum and (**B**) class level. Only phyla or classes with a relative abundance exceeding 1% across all samples are represented. C, coal-mined site; S, seashore-adjacent site. C.te, *C. tetragona*; D.oc, *D. octopetala*; S.ac, *S. acaulis*.

A total of 31 bacterial phyla were identified at the phylum level. The eight most abundant phyla were: Proteobacteria (average 24.0%), Acidobacteria (14.9%), Bacteroidetes (13.9%), Firmicutes (10.7%), Verrucomicrobia (8.0%), Actinobacteria (4.5%), Planctomycetes (2.5%) and Chlamydiae (1.0%) (Fig. 6A). Phyla with a relative abundance < 1% were grouped into “others” and unclassified phyla made up 15.9% of relative abundance. Overall, Proteobacteria was abundant in all samples. This again agrees well with earlier studies of tundra soils [4, 61, 62]. Minor differences were observed between coal-mined site and seashore-adjacent site at the phylum level. Samples from coal-mined site had a relatively higher abundance of Bacteroidetes and Firmicutes; however, Acidobacteria was relatively more prevalent in seashore-adjacent sites. At the class level, bacterial communities were composed mainly of taxa belonging to the classes Alphaproteobacteria (12.0%), Clostridia (8.4%), Bacteroidia (7.0%), Spartobacteria (6.3%), Betaproteobacteria (4.8%) and Actinobacteria (4.4%) (Fig. 6B). The communities from the coal-mined site had an especially high relative abundance of Clostridia and Bacteroidia, except for the bare soil sample. On the other hand, Spartobacteria and Betaproteobacteria tended to be more abundant in the seashore-adjacent site than in the coal-mined site.

Most of the phyla and classes from this study were also observed in a glacier foreland at Ny–Ålesund in previous studies. For one, the relative abundance of Proteobacteria, Acidobacteria, Bacteroidetes, Actinobacteria, and Planctomycetes in this study resembles that found by Kim *et al*. [18]. Also, the dominant phyla (> 5% of total) in soils collected from Adventdalen of Svalbard were Proteobacteria, Actinobacteria, Verrucomicrobia, Acidobacteria and Gemmatimonadetes [63]. Lee *et al*. [52] reported that the most abundant phylum in Ny–Ålesund was Proteobacteria, followed by Actinobacteria, Acidobacteria and Bacteroidetes. Across the Arctic region in Finland, Alaska and Svalbard, soils host a similar relative abundance of Alphaproteobacteria [61]. Geographical variation in microbial community structure, even in Arctic tundra regions, may be due to differences in the formation of the soil layer that led to unique evolutionary processes in the local microbial populations [52, 64]. Also, Massaccesi *et al*. [11] revealed that changes in microbial community structure are driven by the combined effect of plant species and rhizosphere soil characteristics. To improve our understanding of microbial community structure, it is necessary to investigate the spatial heterogeneity of tundra soils, nutrient cycling, temperature variation and soil profiles [52, 65].

### Relationships between the Relative Abundance of Phyla and Soil pH

**Fig. 7:**
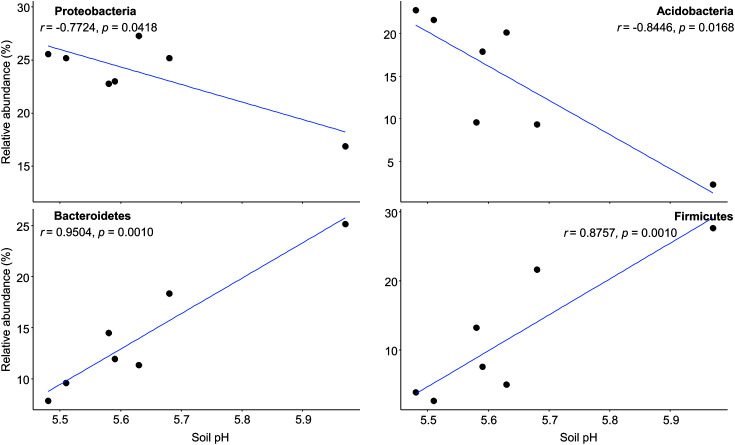
Linear regression results depicting relationships between the relative abundance of four dominant phyla and soil pH. The outlier of bare soil sample from the coal-mined site was excluded before analysis.

The relative abundance of four dominant phyla significantly correlated with soil pH when the bare soil sample outlier was excluded (Fig. 7). Specifically, the relative abundance of Proteobacteria and Acidobacteria decreased toward higher soil pH, whereas that of Bacteroidetes and Firmicutes positively correlated with soil pH. Overall, the bacterial communities in seashore-adjacent samples tended to have more Acidobacteria and lower pH than coal-mined samples (Table 1, Fig. 6). In addition, Acidobacteria had a significantly negative relationship with soil pH (Fig. 7), a result that has been documented by earlier studies [66-69]. The relative abundance of Proteobacteria, which includes Alphaproteobacteria and Betaproteobacteria, decreased with increasing soil pH; however, the results of Shen *et al*. [70] showed a negative relationship between the relative abundance of Alphaproteobacteria and soil pH and a positive relationship between the relative abundance of Betaproteobacteria and soil pH in Chagbai Mountain. Chu *et al*. [68] proposed that a positive relationship existed between soil pH and the relative abundance of Alphaproteobacteria and Betaproteobacteria in the Arctic tundra. Another study showed that the relative abundance of Proteobacteria remained stable over variations in soil pH in the Arctic region [71]. Similar to our results, a strong positive correlation between the relative abundance of Bacteroidetes and soil pH was reported in Arctic soil [ 68, 71]. Although there were differences in the relationships between soil bacteria and pH across the Arctic region, pH was identified as the key environmental driver of structural differences in Arctic soil bacterial communities [71].

### Bacterial Community Diversity and Richness

Bacterial community diversity and richness indices were not significantly different between coal-mined and seashore-adjacent sites (Table 2). Bacterial diversity fluctuated with plant species and site at this small spatial scale. In the coal-mined site, the bacterial community of the *S. acaulis* rhizosphere had lower Shannon’s and Simpson’s indices, whereas the bare soil sample had higher diversity according to all three indices. In contrast, bare soil from the seashore-adjacent site had lower diversity indices than other three samples from plant rhizospheres. Due to the small number of samples, we did not find a clear pattern between bacterial community diversity and plant species or local environment. Soil water content was higher in vegetated soil than in bare soil in Ny–Ålesund, and is the main factor affecting bacterial abundance and soil respiration [72, 73]. Surprisingly, in July 2015, when the survey was conducted, monthly precipitation was only half that of mean precipitation for July. Therefore, this abnormal climate in July 2015 may have affected the soil moisture and microbial community.

Additionally, the variation of diversity indices is partially caused by the proportional abundance of genera in a sample, and these indices are more sensitive toward genus evenness [74]. Lee *et al*. [52] showed that soil bacterial diversity indices as well as the average gene copy number did not significantly differ between organic and mineral layer soil types. Other studies have found evidence for soil microbial community diversity correlating with soil physicochemical properties such as soil pH, C:N ratio, total organic matter, soil moisture, exchangeable Mg^2+^ [13, 52, 70], elevation [70], soluble N and N mineralization potential [56]. However, approximately 50% of the variation in bacterial diversity and structure remains unexplained, although environmental factors have been considered to be a primarily factor controlling soil bacterial distribution [75, 76].

To summarize, we observed that bacterial community composition was more similar within samples of the same local environment than within samples associated with the same plant. We also found that the relative abundance of major phyla (*e.g.*, Proteobacteria, Acidobacteria) correlated with soil pH. In our study, the small number of samples limited statistical power in analyses. Using replicates for each site would allow for a more accurate assessment of bacterial community composition and diversity. Nevertheless, this study provides information that can be used to predict ecological and functional diversity in soil microbial communities in Ny–Ålesund, Svalbard. To improve our understanding of soil bacterial communities in the Arctic, it is necessary to conduct concurrent, long-term monitoring of abiotic factors, such as the spatial heterogeneity of soils, soil temperature, and soil moisture, alongside biotic factors with respect to vegetation type as well as plant and animal species.

## Figures and Tables

**Table 1:** Soil chemical characteristics at sampling sites.

	Coal-mined site				Seashore-adjacent site			
	
	Bare soil	C.te	D.oc	S.ac	Bare soil	C.te	D.oc	S.ac
EC (mS/m)	16.6	8.2	7.4	6.2	3.8	10.6	9.2	14.0
pH	6.44	5.58	5.68	5.97	5.48	5.59	5.63	5.51
OM (%)	12.5	7.9	7.4	5.1	4.3	8.6	6.7	10.6
C (%)	26.1	9.1	9.8	10.8	5.1	15.6	10.7	15.6
N (%)	0.68	0.57	0.57	0.62	0.42	0.82	0.75	1.10
C:N ratio	38.27	15.89	17.1	17.5	12.2	19.1	14.2	14.3
Exchangeable cations (mg/g)								
E-Ca^2+^^*^	6.92	10.60	11.71	7.50	1.74	4.62	3.13	4.99
E-K^+^	0.09	0.14	0.13	0.19	0.08	0.11	0.10	0.18
E-Mg^2+^^*^	0.96	1.39	1.68	0.99	0.39	0.83	0.58	0.78
E-Na^+^	0.05	0.05	0.05	0.02	0.03	0.05	0.03	0.06
Heavy metals (mg/g)								
Cr	0.02	0.01	0.01	0.01	0.02	0.01	0.01	0.01
Cu	0.02	0.01	0.01	0.01	0.01	0.01	0.01	0.01
Fe	17.65	8.90	9.76	7.54	15.58	13.34	9.97	12.54
Mn	0.41	0.10	0.12	0.14	0.21	0.38	0.44	1.19
Pb	0.01	0.00	0.01	0.01	0.01	0.01	0.01	0.01
Zn	0.06	0.03	0.03	0.03	0.05	0.06	0.03	0.06

Asterisks indicate *p* < 0.05 in comparisons between coal-mined and seashore-adjacent sites, as determined by a Mann–Whitney U test. C.te, *C. tetragona*; D.oc, *D. octopetala*; S.ac, *S. acaulis*; EC, Electrical conductivity; OM, organic matter.

**Table 2:** Microbial diversity indices obtained from each sample.

	Coal-mined site				Seashore-adjacent sites			
	
	Bare soil	C.te	D.oc	S.ac	Bare soil	C.te	D.oc	S.ac
Shannon's index	6.6	6.5	6.4	6.1	6.2	6.5	6.5	6.4
Simpson's index	311.7	254.6	283.6	161.9	128.7	195.9	249.0	173.7
Chao1 estimated richness	4225.4	3767.5	3174.7	3402.8	3062.1	3830.6	4316.6	3612.6

C.te, *C. tetragona*; D.oc, *D. octopetala*; S.ac, *S. acaulis*.
